# Prediction of COVID-19 patients’ participation in financing informal care using machine learning methods: willingness to pay and willingness to accept approaches

**DOI:** 10.1186/s12913-024-11250-2

**Published:** 2024-07-10

**Authors:** Vajihe Ramezani-Doroh, Somayeh Najafi-Ghobadi, Faride Karimi, Maryam Rangchian, Omid Hamidi

**Affiliations:** 1 Department of Health Management and Economics, School of Public Health, Hamadan University of Medical Sciences, Hamadan, Iran; 2 Modeling of Noncommunicable Diseases Research Center, Hamadan University of Medical Sciences, Hamadan, Iran; 3 Department of Industrial Engineering, Kermanshah University of Technology, Kermanshah, Iran; 4 Department of Clinical Pharmacy, School of Pharmacy, Hamadan University of Medical Sciences, Hamadan, Iran; 5 Department of Science, Hamedan University of Technology, Hamedan, Iran

**Keywords:** Healthcare Financings, Informal care, Patient Preferences, COVID-19, Machine learning

## Abstract

**Background:**

Informal care plays an essential role in managing the COVID-19 pandemic. Expanding health insurance packages that reimburse caregivers' services through cost-sharing policies could increase financial resources. Predicting payers' willingness to contribute financially accurately is essential for implementing such a policy. This study aimed to identify the key variables related to WTP/WTA of COVID-19 patients for informal care in Sanandaj city, Iran.

**Methods:**

This cross-sectional study involved 425 COVID-19 patients in Sanandaj city, Iran, and 23 potential risk factors. We compared the performance of three classifiers based on total accuracy, specificity, sensitivity, negative likelihood ratio, and positive likelihood ratio.

**Results:**

Findings showed that the average total accuracy of all models was over 70%. Random trees had the most incredible total accuracy for both patient WTA and patient WTP(0.95 and 0.92). Also, the most significant specificity (0.93 and 0.94), sensitivity (0.91 and 0.87), and the lowest negative likelihood ratio (0.193 and 0.19) belonged to this model. According to the random tree model, the most critical factor in patient WTA were patient difficulty in personal activities, dependency on the caregiver, number of caregivers, patient employment, and education, caregiver employment and patient hospitalization history. Also, for WTP were history of COVID-19 death of patient's relatives, and patient employment status.

**Conclusion:**

Implementing of a more flexible work schedule, encouraging employer to support employee to provide informal care, implementing educational programs to increase patients' efficacy, and providing accurate information could lead to increased patients' willingness to contribute and finally promote health outcomes in the population.

**Supplementary Information:**

The online version contains supplementary material available at 10.1186/s12913-024-11250-2.

## Introduction

COVID-19, with its considerable impact on countries, has challenged many health systems worldwide. The pandemic has placed considerable pressure on the limited resources of health systems. There are many examples of rationing hospitalization care for COVID-19 patients [1]. Informal care, which involves providing care by family, friends, and relatives without compensation [2], is potentially essential in managing the COVID-19 pandemic [3]. Governments have the opportunity to utilize potential capacities of informal care to cope with the unpredictable demands of health services. Taking advantage of caregivers' capacities relates to creating policies that can support and enhance their contributions. One possible way to this end is by expanding health insurance packages that reimburse caregivers' services. Designing such insurance packages through cost-sharing policies can increase financial resources. In essence, by sharing the full costs of financing informal services among different financing schemes, such as government schemes and consumers’ out-of-pocket payments, policymakers can ensure sustainable and effective financial resources [4]. Furthermore, cost-sharing policies could serve as an incentive to discourage excessive utilization of healthcare services [5]. Despite the positive effects of consumer payments for healthcare services, there is also a drawback to these policies. Specifically, financial hardships may prevent many patients from accessing healthcare services, [6] underscoring the importance of accurately predicting payers' willingness to contribute when implementing such policies.

In situations where there are markets for goods, it may be feasible to estimate patients' willingness to contribute (the value of services for consumers) by observing their behavior [7]. Since there is no market for informal care, the value of informal care could not be reflected by its price and through markets; in such cases, alternative methods could be used to assess the value of these services. Contingent valuation techniques are one of these methods used to determine the value of non-marketable services [8] and predict consumer's willingness to participate in financing. The contingent valuation method (Willingness to Pay(WTP) and Willingness to Accept (WTA)) has become popular in health economics literature [9]. WTP is the maximum amount that someone would pay to get something, while WTA is the minimum amount that someone needs to give up [8]. Typically, individuals are willing to pay less to acquire something (WTP) than they would demand to give up (WTA).This difference between WTP and WTA is important, and many studies have shown this [10]. Paying attention to this difference would help policymakers decide which interventions are feasible and what outcomes they expect [11]. Furthermore, recognizing the WTP-WTA difference helps allocate resources effectively. It shows the true value of things, which can guide decisions on pricing, subsidies, and distribution of resources [12].

Therefore, both methods are used to determine the level and related factors of patients' cost-sharing policies [13]. Eliciting patients' preferences and identifying their responses to a given pricing policy, which have different characteristics, could help set policies with limited adverse effects on deprived patients groups [14, 15].

Accurate estimation of WTP/WTA and its predictors could facilitate effective policymaking, especially in developing health insurance packages [14, 15]. There are traditional methods for identifying significant variables related to the valuation of informal care through the WTP/WTA approach [16]. Many studies have used traditional regression models (e.g., logistic regression) to predict these variables. These models have many restriction assumptions such as linearity. Recently, machine learning techniques have received considerable attention and have shown promising performance in prediction problems (both regression and classification). These methods do not have many restrictive assumptions faced by traditional models, and are widely used for dealing with traditional restricted assumptions (e.g. nonlinear and complex relationships between response and explanatory variables). Machine learning models can obtain more accurate predictions for response variables. These methods consider a complex relationship between response and explanatory variables, called a “black box”, which usually provides more accurate predictions for the response variable than traditional models. Nevertheless, their performance may vary in different situations, and no model works perfectly in all datasets. Random trees, support vector machine and neural networks are among the most widely used machine learning methods and their accurate prediction has been confirmed in various studies [17, 18].

In Iran, health care system offers a three-level approach. The Ministry of Health operates free basic care clinics at the first level. District centers oversee these clinics and provide additional hospital services. Provincial hospitals address these complex medical cases. The health system combines public and private facilities, with insurance plans to ease costs [19]; however, patients still face high out-of-pocket costs that can be crippling [20]. Despite working towards universal healthcare coverage, challenges such as high costs and inflexibility persist [21]. As Iran moves towards universal health coverage, valuing all services, including non-marketable ones like informal care, is crucial. This helps track progress, identify funding gaps, and ensure fairness for everyone [22]. Additionally, with limited resources, it is crucial to allocate them effectively. Understanding the value of informal care and its determinants sheds light on government subsidies and helps design sustainable funding solutions such as insurance or cost-sharing [23].

However, some studies have used machine learning methods in other aspects of the COVID-19 pandemic [24, 25], and no study has used these methods to examine the critical driving factors in the valuation of informal care for COVID-19 patients. This study aimed to develop predictive models to examine the critical driving factors in the monetary valuation of informal care for COVID-19 patients (through both WTP and WTA approaches) and select the model with better performance in the west of Iran, Sanandaj city. We hope that the results of the present study will provide a better picture and help with better government and insurance policies [14, 15] for future waves of a pandemic.

## Methods

### Participants, study design, and variables

The data was collected in Sanandaj city, west of Iran, between June to September 2021. The population was Sanadaj citizens diagnosed with COVID-19 (with a positive polymerase chain reaction (PCR)) in the month prior to the interview. using the following formula and assuming 10% attrition the sample size was calculated to be 425 patients:

$$n\;=\;\left(\frac{Z1-{\displaystyle\frac\alpha2}\;V}\triangle\right)$$

Where $\Delta\;=$ difference between the real and estimated values of WTP/WTA = 0.1

Z = 1.96.

V = CV = 1.

Respondents were volunteers in participating in the study. They verbally expressed their consent to participate in the study. Data collection involved a telephone interview conducted by trained interviewers with each patient. The patients were randomly selected from the list of individuals diagnosed with COVID-19 during the study period. For patients under 18 years old, their parents answered to the questions. The data was collected through a questionnaire consisting two parts. The first part measured patients' demographic, socio-economic, health status and other characteristics, while the second part assessed their WTP/WTA for informal care.

#### The first part (explanatory variables)

In this part, the following variables were measured. Demographic (sex, age, being households head), socio-economic indicators (education level, employment status, marital status, household size, having health insurance), patients’ health status, patients' underlying diseases, patients' hospitalization history due to COVID-19, patients' perceived difficulty in four activity categories (indoor activities, outdoor activities, personal care, going to formal care centers) during receiving informal care, number of caregivers, patients' perceived dependency to caregiver, patient’s number of hospitalization days, patients' history of COVID-19 infection among relatives, patients' history of COVID-19-related deaths among relatives.

#### The second part (outcomes)

In this section, patients were asked to imagine a government plan supporting informal caregivers' services. Then, patients have been asked to reveal their WTP and WTA for these services through two scenarios. To determine the monetary value of informal care, an open-ended format question was utilized to assess the monetary worth of one hour of informal care. The respondents expressed the maximum (minimum) amount of money they would be willing to pay (receive) for receiving/foregoing one hour more/less care.

The scenario for measuring WTP was “Let's suppose that at the time of your COVID-19 infection, the government had a program to support COVID-19 patients where nurses or other caregivers who had the necessary expertise would take care of you at home. Considering the most challenging activity for you, what was the maximum amount you would be willing to pay from your /your family (if you did not have any income by yourself) income for these caregivers to take care of you for one hour instead of your main caregiver?”. The scenario for measuring WTA was “Let's suppose that at the time of your COVID-19 infection, the government had a plan to pay you cash in exchange for reducing the time your main caregiver spends with you. In this case, considering the most challenging activity for you, how much would you be willing to receive to have one hour less of care from your main caregiver and receive care from other expert caregivers?”.

Since some individuals may reveal a zero amount for WTP/WTA, it is important to distinguish whether these zero responses are real (which arisen from financial constraint) or protest (which arisen from preferences or objections). To identify protest zeros, respondents were asked to specify the reasons behind their zero WTP/WTA. If patients indicated that their zero responses were due to their preferences or objections, it was considered as protest; otherwise it was considered as real. The IBM SPSS modeler 18 was applied for data analysis.

### Data analysis

In this study, the two binary outcomes of interest (i.e. WTP and WTA) were considered as the response/output for the classification problem. The three most widely used machine learning methods of random trees, support vector machine, and neural networks were implemented on the training data set (70% of the data was randomly selected) and the models’ prediction power was assessed using the rest of the data (a testing set including the 30% of the remained data). We applied a tenfold cross-validation strategy to tune the hyper-parameters of the used models over the training set. Finally, for the trained model with the best performance over the test set, the most important variables in predicting the positive WTP and WTA were determined separately using the variable importance index [26, 27].

### Classification models

There are different machine learning models, however, their performance may vary in different situations and there is no model that works perfectly in all datasets. We selected the most widely used machine learning methods, including random trees, support vector machine and neural networks, whose accurate prediction is known and confirmed in various research [17, 18]. The dependent variable was WTP/WTA which was included as binary in the analysis.

**Random Trees (RT)** is a tree-based classification model. This classification method utilizes recursive partitioning to divide training records into parts with similar output field values. This model begins by testing the input fields to detect the best split by reducing the impurity index. In this study, the best result was obtained by considering the number of building models equal to 100 and setting sample size 1. For the tree growth, we tuned the various parameters of the model and the following values were obtained: number of nodes = 10,000, tree depth = 10, and minimum child node size = 10.

**Support vector machine (SVM)** is a classification method that produces an efficient model based on structural risk without restricting nonlinearity and distribution of covariates. This model uses a hyperplane to separate the training data and then classify them based on which side of the hyperplane they are on. The best choice for the Kernel function parameter can help the researchers to find the best result from this model. In our research, the polynomial function with degree 3 was determined as the kernel function for the SVM model with the best performance among others. The regularization parameter was optimized by tuning over the training set (tenfold cross-validation), and the best-obtained value was 15. We used expert mode, and the stopping criteria was set 0.001.

**Neural network (NN)** is a subset of machine learning models that the human brain inspires its name. This model imitates the way that biological neurons signal to one another. The NN consists of three layers; an input layer (including explanatory variables), one or more hidden layers, and an output layer (which is used for prediction of the response variable). Proper setting of this model's parameters effectively results in the best performance. Two of the most commonly used types of NNs are Radial Basis Function (RBF) and the Multilayer Perceptron (MLP). We utilized MLP by considering one hidden layer for both responses.

### Implementation, tuning parameters and performance criteria

The models' performance was evaluated by their accuracy, specificity, sensitivity, and the negative and positive probability ratio**.** Then, total accuracy, sensitivity, specificity, positive likelihood ratio, and negative likelihood ratio were calculated by using the following formulas.

$$Sensitivity\;=\;\frac{TP}{TP+FN}\;Specificity\;=\;\frac{TN}{TN+FP}\;Total\;Acuraccy\;=\;\frac{TP+TN}{TP+FP+TN+FN}$$

$$Positive\;likelihood\;ratio\;=\frac{Sensitivity\;}{1-Specificity}$$

$$Negative\;likelihood\;ratio\;\frac{1-Sensitivity}{Specificity}$$

Where TP, FP, TN, and FN are: the number of true positives, false positives, true negatives, and false negatives, respectively.

Finally, the best performing model was used to identify the significant predictors associated with WTP/WTA of COVID-19 patients for informal care.

## Results

### Data description results and variable importance

#### Data description results

Demographic and summary statistics of variables were shown in Table 1. Most patients reported a zero value for WTP and WTA (63% and 66%, respectively). There was no protest response for WTP/WTA. The main reason for having a zero WTP/WTA was financial barriers. The results showed that most of the people who reported a positive amount for WTA and WTP were married women and married men who were covered by social security and had a history of COVID-19 in their relatives. For WTA > 0, 57.5% of their relatives had no history of COVID-19 death. This is also 70.3% for WTP > 0. Overall, 54.8% of WTA > 0 did not have an academic degree and 53.4% of them were unemployed. The majority of WTP > 0 were employed (58.2%) with an academic degree (51.9). Results revealed that 78% of WTA > 0 and 75.3% of WTP > 0 had no a history of hospitalization. Most of the patients' caregivers were their children or spouses (78.1% and 72.8% for WTA > 0 and WTP > 0, respectively). More information was shown in Table 1.

**Table 1:** Summary statistics of the characteristics of the patients

**A) Categorical variables**																
Variables		**WTA**					**Variables**					**WTP**				
		**No**		**Yes**								**No**		**Yes**		
		**n**	**%**	**n**	**%**							**n**	**%**	**n**		**%**
Patient sex							Patient sex									
Man		141	50.5	72	49.3		Man					132	49.4	81		51.3
Woman		138	49.5	74	50.7		Woman					135	50.6	77		48.7
Marital status of patient							Marital status of patient									
Single		84	30.1	40	27.4		Single					72	27	52		32.9
Married		195	69.9	106	72.6		Married					195	73	106		67.1
Patient morbidity to underlying diseases							Patient morbidity to underlying diseases									
No		178	63.8	104	71.2		No					174	65.2	108		68.4
Yes		101	36.2	42	28.8		Yes					93	34.8	50		31.6
Patient health status							Number of household members of patient									
Lower than moderate		90	32.3	55	37.7		=<3					122	45.7	95		60.1
Greater than moderate		189	67.7	91	62.3		>3					145	54.3	63		39.9
Type of health insurance of patient							Type of health insurance of patient									
No		21	7.5	4	2.7		No					21	7.9	4		2.5
Social security		147	52.7	79	54.1		Social security					134	50.2	92		58.2
Other		111	39.8	63	43.2		Other					112	41.9	62		39.2
History of COVID-19 infection of patient's relatives							History of COVID-19 infection of patient's relatives									
No		24	8.6	13	8.9		No					28	10.5	9		5.7
Yes		255	91.4	133	91.1		Yes					239	89.5	149		94.3
History of COVID-19 death of patient's relatives							History of COVID-19 death of patient's relatives									
No		156	55.9	84	57.5		No					129	43.8	111		70.3
Yes		123	44.1	62	42.5		Yes					138	51.7	47		29.7
Number of household members of patient							Relation care giver									
<=3		142	50.9	75	51.4		Spouse or child					183	68.5	115		72.8
>3		137	49.1	71	48.6		Other					84	31.5	43		27.2
Relation care giver							Patient health status									
Spouse or child		95	34.1	114	78.1		Lower than moderate					81	30.3	64		40.5
Other		184	65.9	32	21.9		Greater than moderate					186	69.7	94		59.5
Number of caregivers							Number of caregivers									
1		187	67	108	74		2					195	73	100		63.3
>=2		92	33	38	26		=<3					71	27	58		36.7
Patient difficulty in personal activities							Patient difficulty in personal activities									
No		181	64.9	127	87		No					183	68.5	125		79.1
Yes		98	35.1	19	13		Yes					84	31.5	33		20.9
Patient difficulty in indoor activities							Patient difficulty in indoor activities									
No		125	44.8	73	50		No					134	50.2	64		40.5
Yes		154	55.2	73	50		Yes					133	49.8	94		59.5
Patient difficulty in outdoor activities							Patient difficulty in outdoor activities									
No		110	39.4	57	39		No					109	40.8	58		36.7
Yes		169	60.6	89	61		Yes					158	59.2	100		63.3
Patient difficulty in going to formal care centers							Patient difficulty in going to formal care centers									
No		129	46.2	71	48.6		No					131	49.1	69		43.7
Yes		150	53.8	75	51.4		Yes					136	50.9	89		56.3
Patient education							Patient education									
Without academic degree		165	59.1	80	54.8		Without academic degree					169	63.3	76		48.1
With academic degree		114	40.9	66	45.2		With academic degree					98	36.7	82		51.9
Patient employment							Patient employment									
Unemployed		178	63.8	78	53.4		Unemployed					190	71.2	66		41.8
Employed		101	36.2	68	46.6		Employed					77	28.8	92		58.2
Dependency to caregiver							Dependency to caregiver									
Less than very much		132	47.3	81	55.5		Less than very much					132	49.4	81		51.3
Completely or very much		147	52.7	65	44.5		Completely or very much					135	50.6	77		48.7
Patient hospitalization history							Patient hospitalization history									
No		172	61.6	114	78.1		No					167	62.5	119		75.3
Yes		107	38.4	32	21.9		Yes					100	37.5	39		24.7
Education of caregiver							Marital status of caregiver									
Without academic degree		167	59.9	93	63.7		Single					52	19.5	34		21.5
With academic degree		112	40.1	53	36.3		Married					215	80.5	124		78.5
Marital status of caregiver							Caregiver employment									
Single		55	19.7	31	21.2		Unemployed					174	65.2	86		54.4
married		224	80.3	115	78.8		employed					93	34.8	72		45.6
Caregiver employment							Caregiver education									
Unemployed		162	58.1	98	67.1		Without academic degree					173	64.8	87		55.1
Employed		117	41.9	48	32.9		With academic degree					94	35.2	71		44.9
Total		279	0.66	146	0.34		Total					267	0.63	158		0.37
**B) Continuous variables**																
**Variables**		No							Yes							
		N	Min	Max		Mean		Std. dev	N	Min	Max		Mean		Std. dev	
**WTA**	Patient age	279	12	87		43.56		15.46	146	18	80		41.81		13	
	Caregiver age	279	18	75		40.06		11.76	146	18	72		41.07		11.38	
WTP	Patient age	267	12	87		43.29		15.12	158	12	80		42.39		13.90	
	Caregiver age	267	18	75		39.75		11.33	158	18	72		41.53		12.07	

#### Model comparison

The results of comparison based on the mean and standard deviation of total accuracy, sensitivity, and specificity for all models are represented in Table 2. The total accuracy of all models was greater than 0.70, nevertheless, this criterion the random tree model achieved the largest total accuracy among others (0.95 in predicting WTA and 0.92 in predicting WTP) followed by the SVM (0.87 in predicting WTA and 0.78 in predicting WTP). Also, in the random trees model, sensitivity was higher than other models (0.91 in predicting WTA and 0.87 in predicting WTP). Moreover, the specificity of random trees model was greater than 0.9 (0.93 in predicting WTA and 0.94 in predicting WTP) which was comparable with that of the neural network model (0.95) in predicting WTA and it was better than those of the support vector machine and neural networks models in predicting WTP.

**Table 2:** The performance of three classification models in predicting patients’ WTA and WTP over the test set

**Outcome**	**Models**	**Total accuracy**		**Sensitivity**		**Specificity**		**Positive likelihood ratio**		**Negative likelihood ratio**	
		Mean	Std. dev	Mean	Std. dev	Mean	Std. dev	Mean	Std. dev	Mean	Std. dev
**WTA**	**Random trees **	0.95	0.008	0.91	0.006	0.93	0.003	∞	∞	0.193	0.039
	**Support vector machine**	0.87	0.008	0.64	0.071	0.87	0.008	10.48	2.3	0.447	0.201
	neural **network**	0.70	0.007	0.42	0.009	0.95	0.001	∞	∞	0.819	0.646
**WTP**	**Random trees**	0.92	0.003	0.87	0.006	0.94	0.002	∞	∞	0.19	0.038
	**Support vector machine**	0.78	0.017	0.65	0.018	0.82	0.016	∞	∞	0.58	0.40
	neural **network**	0.73	0.007	0.54	0.014	0.86	0.014	4.31	6.79	0.785	0.557

#### Variable importance

**Fig. 1:**
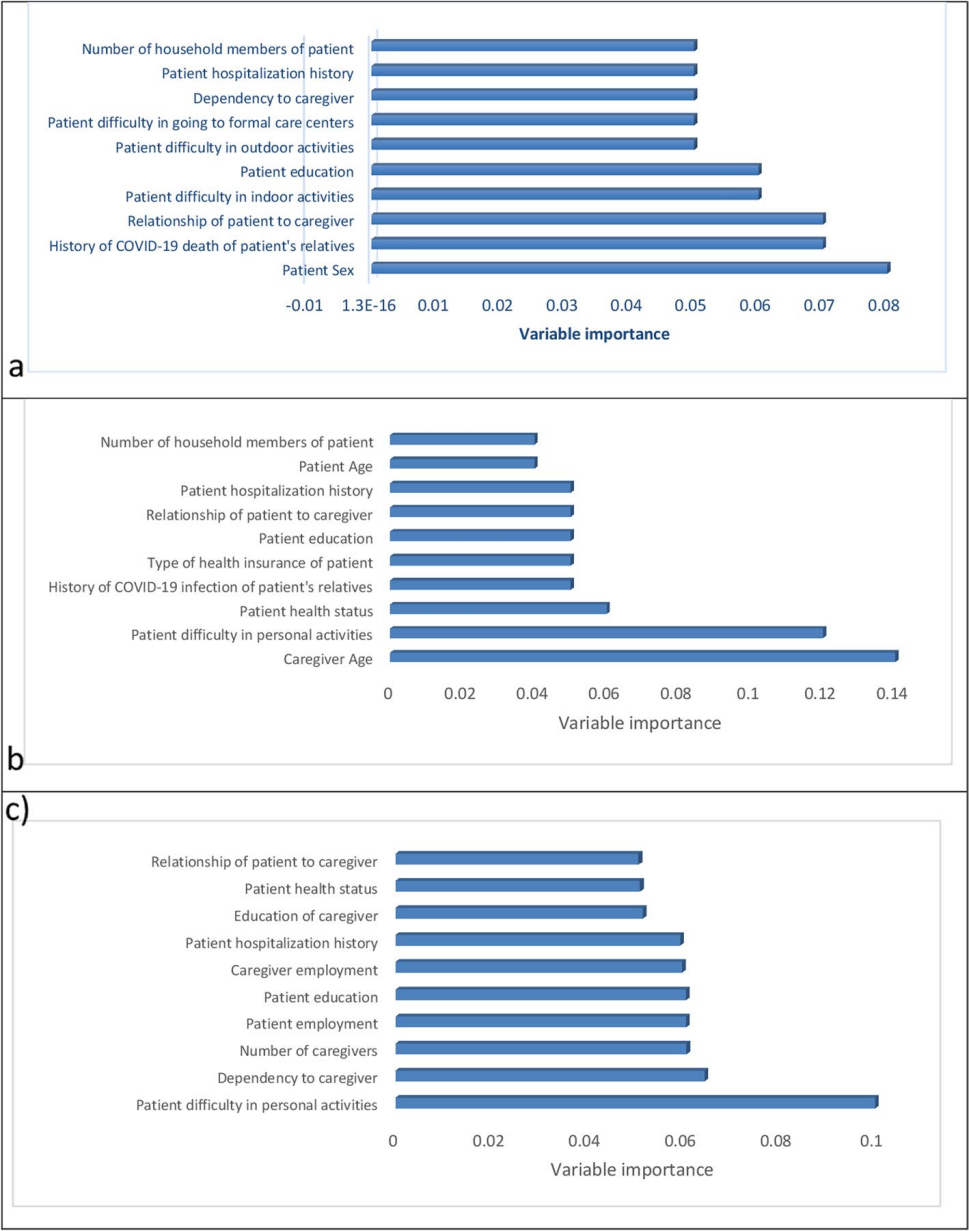
Variable importance of various data mining models in predicting WTA (a: SVM; b: NN; c: RT)

**Fig. 2:**
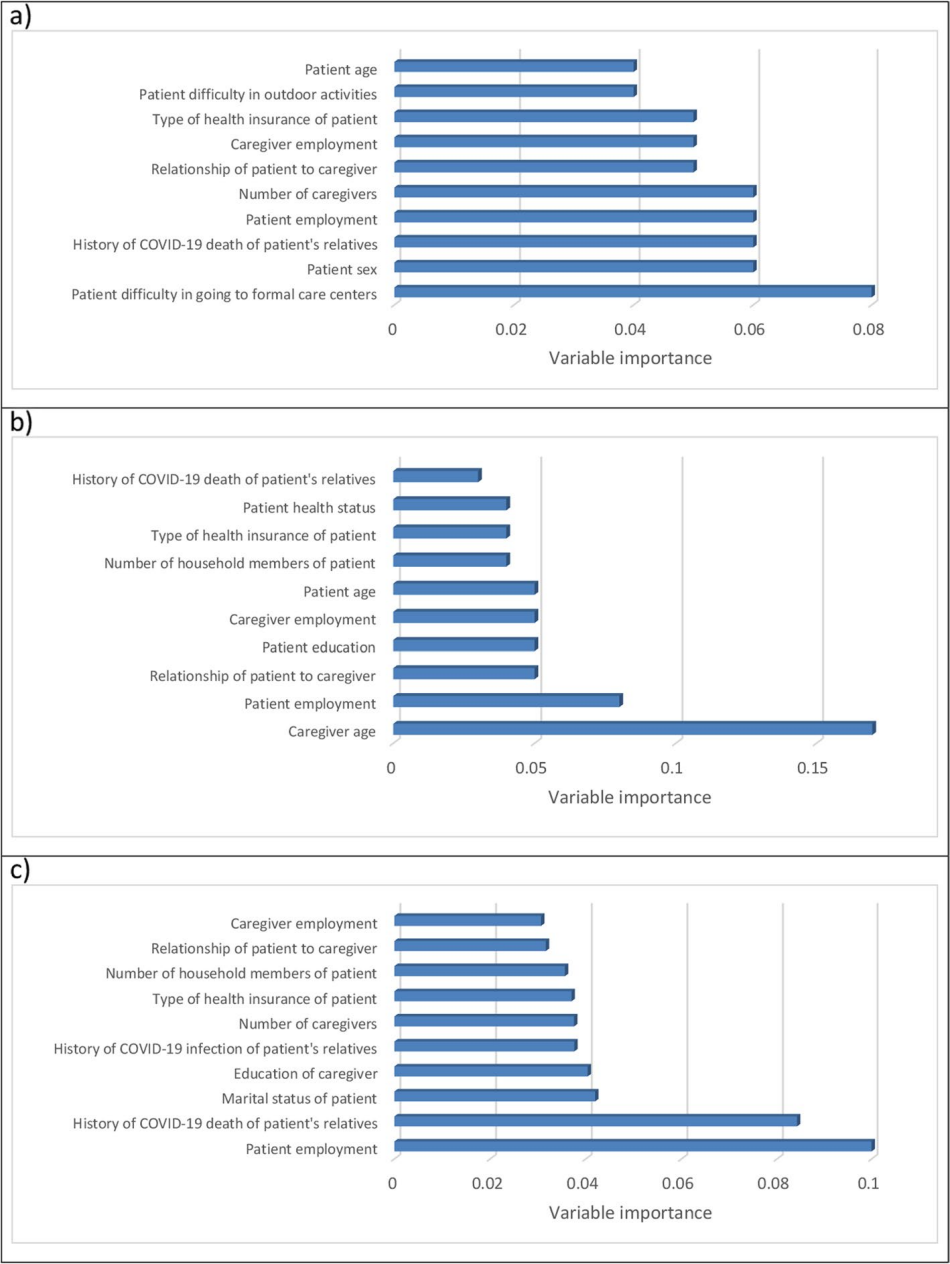
Variable importance of various data mining models in predicting WTP (a: SVM; b: NN; c: RT)

The variable importance of the data mining models used in predicting WTA and WTP was shown in Figs. 1 and 2. Based on random trees, variables with importance greater than 0.05 for patient’s WTA were patient difficulty in personal activities, dependency to caregiver, and number of caregivers, patient employment, patient education, caregiver employment, and patient's hospitalization history. The use of random trees for the patient’s WTP, the patient employment, and the history of COVID-19 deaths of the patient’s relatives were factors whose importance was greater than 0.05.

## Discussion

This study aimed to investigate factors influencing patients' valuation of informal care through machine learning techniques. Patients' preferences are potentially very important in setting effective insurance policies. The findings of this study indicated that the RT model demonstrated good performance, highlighting key variables in patients' valuation using two different methods (WTP and WTA).

Based on the RT model, the most critical factor in patients' decision for payment (WTP) was their employment status. Concerning WTA, this variable was ranked fourth in importance for patients. Having a regular source of income could facilitate and ensure individual’s participation in following their treatment, as several studies have indicated that patients from economically advantaged groups in terms of employment [28] or income [29]were more inclined to contribute to their treatment programs. In this study, employment status served as a proxy for payment capability; due to significant amount of missing data on patients' income, we did not include this variable in our analysis. Employment status may be related to a higher opportunity cost for employed individuals compared to unemployed individuals, shaping their willingness to contribution. As Legese et al. demonstrated that patients with a permanent job or self-employment tended to value informal care more. However, those in temporary positions showed a decreased value of informal care. Moreover, individuals with paid work experience put significantly more value on informal care [30]. Being absent from work could result in a higher costs for employed individuals. To mitigate these costs, groups of workers might show a greater willingness to pay to return to work sooner. Restrictions in the workplace, such as a limit on available leave days, could hinder these groups and impact how they value services monetarily. During crises like COVID-19, leveraging community resources could be made easier with a flexible work environment and employer support to extend leave, ensuring optimal health for patients. Surprisingly, a study on cancer patients revealed that those in professional jobs had a lower WTP for home-based blood transfusion [31]. In terms of WTA, Patients' job status emerged as the fourth predictor. In this study, employment could serve as a proxy for respondents' economic status, influencing how they respond to financial incentives based on their social and economic position. This aligns with findings from other studies, indicating varying responses to financial incentives based on different economic classes. Notably, there was a non-linear relationship between patient income and willingness to accept. Notably, there was a non-linear relationship between patient income and WTA [32]. De Meijer et al. also emphasized the crucial role of patients' financial capacity in their WTA. In their study, the only significant factor affecting patients' WTA was a care budget. Patients with a care budget demonstrated a higher WTA in exchange for forgoing informal care [33].

Patients' perceived risk in terms of the history of relative deaths from COVID-19 ranked second among determinants of WTP. Numerous studies have highlighted the positive impact of perceived risk on patients' healthcare valuation decisions [34]. Another factor reflecting patients' concerns about COVID-19 was the history of relatives' morbidity to COVID-19, which ranked fifth in the RT model. Previous studies have also emphasized the influence of concerns about the severity of the disease on individuals' valuation. Chaugule et al. discovered that experiencing treatment side effects reduced the odds ratio of WTP among hemophilia patients [35].

Variables indicating the severity of illness (such as patients' difficulty in performing personal activities and their dependency on caregivers) were the most crucial factors in determining monetary acceptance. In terms of WTA, patients' difficulty in doing personal activities emerged as the most significant variable in patient valuation. While the direction of the relationship between patient WTA and this variable remains unclear, it is reasonable to expect that patients facing less difficulty with personal activities would be more willing to accept money. De Meijer et al. found a negative association between recipient functional ability and their WTA [33]. Another study on tuberculosis patients revealed that those struggling to adhere to treatment follow-ups were more likely to respond positively to monetary incentives and accept money [32]. An essential characteristic of patients is their ability to perform regular tasks without fear of adverse events [36]. Patients' ability to do their tasks could be assessed by their health status, and interventions aimed at supporting the most severely ill patients could be beneficial. Allocating scarce healthcare resources to the most disadvantaged patients may increase health system efficiency and equity. The level of dependency on the caregiver ranked as the second most crucial variable in WTA. Patients' dependency on caregivers has been well-documented. Van den Berg et al. observed a negative correlation between patients' health status and their WTA [16]. De Meijer et al. also observed that patients' WTA did not vary based on their need for assistance in the organizational task [33]. Floyd et al. found that a higher perceived need in patients with chronic knee condition resulted in a more WTP [37]. However, a study in type2 diabetes patients indicated that the severity of patients' morbidity was not significantly associate with patients' WTP for an SMS plan related to their disease [38].

The number of caregivers identified as the third variable defining the patient's inclination to accept money. It is rational to expect that by increasing in the number of caregivers, patient' WTA will decrease, as they may rely on other caregivers if their primary one cannot provide care. Surprisingly, a study by Legese et al. showed that by increasing in the number of external caregivers, patients placed a higher value on informal care [30]. Another study in Singapore revealed that reducing the caregivers' burden was not patients' priority; except for those with cancer [39]. Access to cheap substitute caregivers could shape the results of Malhorta et al. [40]. Some studies have found that by increasing the hours of unpaid work may lead to more unmet need in care recipients [41], indicating that an increase in the caregivers' number necessarily may not be related with a better level of meeting patients' needs and consequently lower probability of accepting money for forgoing informal care. Van den burg et al. also found that patients' WTA did not significantly change by caregivers' health status [16], which could be related to the number of caregivers.

Education is an essential factor in evaluating health care services and health status [29, 38, 42, 43]. Lieu et al. assessed the value of QALY in two distinct groups (patients and community members). They found that the mean of WTP was significantly higher among educated patients [29]. Isah et al. pointed out that there was a positive relationship between patients' education level and their WTP for the prevention of mother-to-child transmission (PMTCT) drugs. However, regarding their WTA, this study showed that for PMTCT treatment and support and therapeutic intervention, there was a negative relationship [43]. Some studies did not found a significant relationship between patients' education and their valuation [44, 45]. Augusti et al. observe no significant association between educational status and WTP in patients with dental problems [45]. In this study, patients' education ranked fifth in importance when evaluating patients' WTA for informal care, although in the WTP approach this variable had importance less than 0.05. The perceived advantage of receiving care from educated caregivers could explain the impact of patients' education on their monetary valuation. Maybe educated patients had more unmet needs and were more willing to seek care from certified caregivers. A study by Fautrel et al. in Canada revealed that patients' perception of treatment benefits plays an essential role in their valuation of the treatment plan. The authors claimed that patients with the moderate disease preceive considerable benefits in treatment [44]. Patients’ opinion on the health system was one of the critical variables in Fautrel et al. study, which increased the odds ratio of WTP for patients enrolled in the public program. It is plausible that educated participants in the current study held a positive view of receiving informal care from the formal healthcare system. More perceived dangerous consequences of COVID-19 may be another explanation for the effects of education on WTP. As health status for educated patients may have more importance due to their higher perceived risk. As Augusti et al. showed, patients who cited high importance for their oral care were more willing to pay for their treatment [45]. Another study by De Meijer et al. did not report any statistically significant relationship between patients' education level and their WTA/WTP [33].

Caregiver employment was another important variable for reporting a positive WTA. As previously mentioned, limitations in the work place could influence how patients value informal care. Probably these limitations could convince patient to seek care from other providers instead of their caregiver.

Patient hospitalization history was the last important variable in reporting a positive WTA. Experiencing formal services in a specialized setting could impact how patients value informal care.

While this study, to the best of our best knowledge, was the first to evaluate the shaping factors in the monetary valuation of informal care for COVID-19 patients using machine learning models; some limitations should be considered for future planning. The patients were from a city in Iran that has specific cultural and social characteristics, limiting the generalizability of our findings to other regions. The study was conducted during the summer of 2021, and the disease wave at this time differed from other waves. To have an accurate picture of the COVID-19 economic burden, it is essential to repeat this study for other disease variants, as each variant has specific characteristics.

## Conclusions

The perceived value of—of informal care by COVID-19 patients could provide invaluable insights for policymakers. Designing effective supporting plans within the health system, such as expanding insurance coverage for non-marketable services such as informal care, may enhance patient outcomes and decrease the health system burden in times of excessive demand for health care services. The most important variables for patients' involvement in their monetary valuation were their employment status and their health condition. Enabling more flexible work schedules, encouraging employer to support employee to provide informal care, and implementing educational initiatives to increase patients' efficacy and provide accurate information could increase patients' willingness to contribute and promote health outcomes in the population.

### Supplementary Information

Supplementary Material 1.

## Data Availability

The datasets generated during and analyzed during the current study are not publicly available due to the legacy Hamadan University of Medical Sciences restrictions on public sharing data, but are available from the corresponding author upon reasonable request.
