# Supplementary material for: Prediction of COVID-19 patients’ participation in financing informal care using machine learning methods: willingness to pay and willingness to accept approaches
Source: BMC Health Serv Res. 2024 Jul 10;24:796. doi: 10.1186/s12913-024-11250-2 (PMC11234787; doi:10.1186/s12913-024-11250-2)
Supplement: Supplementary file 1 — Supplementary Material 1. [file 12913_2024_11250_MOESM1_ESM.docx]

**Part1: information about the patient and the main caregiver**

Patient questions:

1. **How old are you? ... year**

**2) What is your gender?** Woman man

**3) What was the status of your marriage at the time of getting COVID-19?**

a) Single b) divorced or deceased spouse

c) Married

**4) What is your education?**

a) Illiterate b) less than high school c) High school

d) Associated degree e) Bachelor's degree or higher

**5) What was your employment status at the time of getting COVID-19?**

a) Employed b) retired c)unemployed d)studying e) housewife

**6) When you were infected with COVID-19, were you the household's head?**

Yes No

**7) When you were infected with COVID-19, how many was your household size?** …. Person/s

**8) When you were infected withCOVID-19, how do you evaluate your health status?**

Very good good moderate bad very bad

**9) When you were infected withCOVID-19, which of the following diseases were you suffering from?**

a) Cardiovascular disease

b) Respiratory disease (except for corona complications)

c) Diabetes

d) Cancer

e)Other diseases: please name...

f) I was not suffering from any disease

**10) When you were infected withCOVID-19, did you have health insurance?**

Yes no

**11) If your answer to the previous question is yes, what was your type of insurance?**

a) Social Security Insurance b) Armed Forces Insurance

c) Health Insurance d) other insurances: Please name...

**12) Has anyone of your relations been infected with COVID-19?**

Yes no

**13) Has anyone of your relations died due to COVID-19?**

Yes no

**14) When you were infected withCOVID-19, how many people were taking care of you?** .... Person/s

Now, consider your main caregiver and respond to the following questions:

**15) How old was your main caregiver? ... years**

**16) What is the gender of your main caregiver?**  Woman man

**17) When you were being cared for due to COVID-19, what was the marital status of your main caregiver?**

a) Single b) divorced or deceased spouse

c) Married

**18) What is the education of your main caregiver?**

a) Illiterate b) less than high school c)High school

d) Associated degree e) Bachelor's degree or higher

**19) What is your relation to your main caregiver?**

a) My Child b) My mother/father

c) My husband/wife

d) My brother/sister e) My Friend f) My relative : Please name...

g) Other : Please name...

**20) When you were being cared for due to COVID-19, what was the employment status of your main caregiver?**

a) Employed b) retired c) unemployed d)studying e) housewife

**21) When you were being cared for due to COVID-19, for every following activities how much did you depend on your main caregiver?**

a. Personal activities (such as taking medicine, bathing, eating, accompanying to use the toilet, going up and down the stairs, getting in and out of bed)

Completely very much moderate a little at all

b. Indoor activities (such as washing clothes, cooking, cleaning the house, doing things related to your family members)

Completely very much moderate a little at all

c. Outdoor activities (such as shopping, getting needed medicines, doing banking and administrative work, etc.)

Completely very much moderate a little at all

d. Going to formal care centers that have provided formal care (hospital, clinic, doctor's office)

Completely very much moderate a little at all

**22) When you were infected with COVID-19, totally how much did you depend on your main caregiver?**

Completely very much moderate a little at all

**23) When you were infected with COVID-19, did you hospitalize at hospital?**

No Yes …. Day/s

**Part2: patient's WTP and WTA**

**24) When you were being cared for due to COVID-19, what was the most difficult activity for you to do?**

1. Personal activities
2. Indoor activities
3. Outdoor activities
4. Going to formal care centers that have provided formal care

**25) Let's suppose that at the time of your COVID-19 infection, the government was implementing a program to support COVID-19 patients in which nurses or other caregivers who had the necessary expertise would take care of these patients at home. Considering the activity that was most difficult for you to do (among the activities mentioned in the previous question), what was the maximum amount you would be willing to pay from your income /your family (if you did not have any income by yourself) to have these caregivers take care of you for one more hour instead of your main caregiver? ............................. Toman**

**26) If you were not willing to pay any amount for the mentioned plan, which of the following options is the reason? (You can choose more than one option)**

a- It was unfair that the government asked me to pay money, the government itself should have paid the money

b- I could not afford to pay

c- Taking care of me was moral and human responsibility of my main caregiver and I was not willing to pay for it

d- I think my main caregiver could take care of me better than others

e- Other reason/s (please mention).....

**27) Let's suppose that at the time of your COVID-19 infection, the government was implementing a plan in which it was supposed to pay you an amount in cash in exchange for reducing the time your main caregiver take care of you. In this case, considering the activity that was the most difficult for you to do (among the activities mentioned in question 23), how much would you be willing to receive to be cared for one hour less and do that activity yourself? ............................. Toman**

**28) If you were not willing to receive any amount for the mentioned plan, which of the following options is the reason? (You can choose more than one option)**

a) Taking care of me was moral and human responsibility of my main caregiver/me and it was unfair for me to receive money from the government for taking care of myself

b) I had enough money and I did not need to get money from government

c) I think my main caregiver could take care of me better than me

d) I did not have enough strength (physically and mentally) to take care of myself and I could not do that activity even in exchange for money

e) Other reason/s (please mention).....
